# The Risk of Extreme Streamflow Drought in the Polish Carpathians—A Two-Dimensional Approach

**DOI:** 10.3390/ijerph192114095

**Published:** 2022-10-28

**Authors:** Katarzyna Baran-Gurgul

**Affiliations:** Department of Geoengineering and Water Management, Faculty of Environmental and Power Engineering, Cracow University of Technology, Warszawska 24, 31-155 Cracow, Poland; katarzyna.baran-gurgul@pk.edu.pl

**Keywords:** streamflow drought, hydrological drought, probability distribution, copula function, return period, spatio-temporal characteristic of drought, Polish Carpathian

## Abstract

Poland has relatively small water resources compared to other European countries. Droughts are a characteristic feature of the Polish climate; however, recent years have been particularly warm, causing longer and more severe droughts, including streamflow droughts. The most unfavourable streamflow droughts, considering the economic or social (including health-related) consequences, are the longest and/or the ones with the largest volumes. Such prolonged and severe droughts may constitute a natural disaster threatening public health. The main aim of this article was to define the spatial variability of the annual maximum streamflow drought in the Polish Carpathians and the risk of the maximum streamflow drought of a duration and volume exceeding the given value occurring in this region. This was conducted based on a 30-year time series of daily flows in selected gauging cross sections on rivers in the Polish Carpathians. One- and-two-dimensional probability distributions (utilising a copula function) of the two most important maximum streamflow drought characteristics were identified, specifically duration and volume, which, in consequence, led to identifying the maximum streamflow droughts of a given return period (a given risk level). Maps of maximum streamflow drought hazard were developed and understood as spatial distributions of the maximum streamflow drought frequency of duration and volume exceeding the annual given values. Analysis of the maps allowed for the selection of areas/basins being more or less at risk of extreme annual streamflow drought of a duration and/or volume exceeding the given value.

## 1. Introduction

Drought is a natural climate characteristic which means a deficit or lack of water within an environment, being an inconvenience to people [1,2]. Drought is a complex and multifaceted process in time and space which eludes a clear and objective definition. Beran and Rodier [3], as well as the Flow Regimes from International Experimental and Network Data (FRIEND) research team [4] define drought as a continuous regional event characterised by deviation from the normal conditions of precipitation, humidity, groundwater levels, or river flows.

According to Polish law, drought as a natural disaster threatening the life or health of a large number of people, affecting large scale property or large areas of natural environment is defined by Art. 3, sec. 1, item 1 of The Act of 18 April 2002 on the State of Natural Disaster [5].

The development of drought into its extreme consequences is most often described as a process encompassing four stages (types of droughts): meteorological, agricultural, hydrological, and socioeconomic drought [6,7]. These stages are not disjointed time intervals. This traditional definition of drought based on the abovementioned drought stages view drought through a human-centric lens [8]. These indices can reflect the hydro–meteorological elements that affect the ecosystem, but they do not characterize the role of the ecosystem in the drought evolution. Therefore, some newer research studies consider another class called ecological drought. For example, the Ecological Drought Working Group, established by the Science for Nature and People Partnership (SNAPP) in 2016, defined ecological drought as episodic deficit in available water induced by climate and human factors during the vegetation growth period, which ultimately affects other systems. On this basis, Crausbay et al. [8] defined ecological drought as an episodic deficit in water availability that drives ecosystems beyond thresholds of resilience into a vulnerable state, impacts ecosystem services, and triggers feedback in natural and/or human systems. However, there remains no widely accepted drought index to monitor ecological drought. In this paper, the classical definition of drought was considered.

Prolonged lack, or a significant deficit, of precipitation constitutes the first stage of drought, known as atmospheric or meteorological drought. Maidment [9] defines meteorological drought as a period of time (stated in months or years) during which the supply of moisture to a given area falls below the normal level of moisture supply in a given climate.

Usually in the summer when there is a shortfall of precipitation for a long time and the air temperature remains high, it is likely for intense evaporation of the water retained in soil and surface reservoirs to occur [10]. This increase of the evaporation intensity causes the drying out of the surface first, and then the deeper soil layers. Soil moisture decreases, and the root zone experience water deficit which may cause vegetation to die down. This state causes the atmospheric drought to develop into soil drought, also known as agricultural drought. Wilhite and Glanz [11], as well as Maidment [9] define agricultural drought as a period of time during which soil moisture is insufficient to meet the water needs of plants and sustain normal farming activity.

Hydrological drought is the next stage, after the agricultural one [9,10,11,12]. Usually, potential short rainfalls will not have supplied the underground reservoirs because they will have been absorbed and retained by the ground in full. Dried up and hard soil is not able to receive precipitation of such high intensity, therefore rainwater runs down the surface of the ground with minimal infiltration and not supplying soil retention. Only prolonged precipitation may replenish soil moisture deficiencies. Further extension of a period without precipitation triggers the third stage of the drought process, namely hydrological drought.

Hydrological drought manifests itself in lowered groundwater levels in wells (groundwater drought) and the decreasing of water supply to rivers. When these resources are not recharged by infiltration, but rather depleted by supplying the surface watercourses, they diminish even further. Dębski [10] observes that the state of depleting the groundwater resources at this stage depends on the duration of drought. If it starts the in the early summer months, the depleting of the groundwater resources is prolonged, and may continue until the autumnal rainfalls, or, in the case that these do not occur, even until the winter thaw. If the start of this stage falls in late summer, the depleting of the groundwater resources is shorter, therefore it is not as large.

With the further lack of precipitation, the next stage of the drought process begins, namely streamflow drought. Streamflow drought is most often defined as a continuous period during which streamflow at a given river cross section is below the assumed threshold value of the flow [4,10,12]. This study discusses streamflow drought only, further referred to as drought.

The most unfavourable droughts, considering the socioeconomical consequences, as well as the most interesting ones from the practical perspective, for example in view of assessing the risk of a maximum drought occurring in a given cross section, are the longest droughts and/or the ones with the largest volumes. Such droughts are known as maximum [13,14] or extreme [1,15] droughts. This study discusses two annual maximum droughts: of the longest annual duration *T_max_*, and with the largest annual volume *V_max_*. The selection of these droughts allows for the calculation of the frequency (probability) of occurrence of a maximum drought with a given duration or volume.

Drought duration and volume are correlated, therefore more and more often these variables are described by a bivariate distribution. Bi- or even trivariate probability distributions of drought characteristics were applied in Poland as early as the 1960s; In her research, Zielińska [16,17] used the normal distribution which, due to its symmetry, allows for a good fit to observed data only in some cases. Jakubowski [14], and Węglarczyk and Baran-Gurgul [18] found that joint probability distributions of drought duration and volume may be described using the bivariate lognormal distribution.

Many authors, especially in the most recent studies, recommend using the copula which enables constructing a multivariate distribution based on any univariate marginal distributions. The most commonly applied copulas of drought duration and volume are: The Clayton copula, Plackett copula, Gumbel copula, Frank copula, and Gumbel–Hougaard copula [19,20,21,22,23,24,25,26].

Song and Singh [20] used the Plackett copula to describe drought duration, volume, and the interval between subsequent droughts, whereas Jakubowski [27] used the Gumbel–Hougaard copula to describe drought duration, volume, and the minimum drought discharge. Jakubowski [28,29] concluded that the bivariate generalised Pareto distribution may be used to describe the joint probability distribution of the maximum drought duration and volume, and also a distribution based on the Gumbel–Hougaard copula with generalised extreme value (GEV) distributions as marginal distribution may be used for the same purpose [28]. Tosunoglu and Kisi [30] modelled joint *T_max_* and *V_max_* distribution in Turkey and found that the best one out of the four applied Archimedean copulas (Ali-Mikhail-Haq, Clayton, Frank and Gumbel–Hougaard) was the Gumbel–Hougaard copula. Depending on the gauging cross section, they assumed different marginal distributions (for volume–exponential or Weibull, for duration–Weibull or logistic).

Poland has relatively small water resources, with the ratio of the annual mean river discharge to the population being approximately 1500 m^3^person^−1^year^−1^ and is three times lower compared to Europe’s water resources [31].

Extreme weather events such as droughts, floods, hurricane winds, or torrential rainfalls are characteristic of the Polish climate. In recent years, it can be observed that the number of extreme weather events, including droughts, has been on the increase. The most prone to the adverse effects of droughts resulting from insufficient water resources are those sectors of the economy which rely on water, namely agriculture, water management, energy production, or forestry. Indirectly, however, drought also affects other industries, such as tourism and various other forms of outdoor leisure. Owing to the reduced amount of snow and river flows, drought has direct effects on water sports (boats, kayaking, or swimming) as well as winter sports, such as skiing; drought may result in shorter or shifted seasons for doing these sports [32].

Droughts may also affect public health by causing the drinking water resources to diminish not only in quantity but also in quality. During a drought, the amount of dust particles suspended in the air increases, which degrades the quality of air, and long-term, increases the risk of allergies and respiratory diseases [33].

According to the World Meteorological Organisation, 2015 was the warmest year on record since 1961 [34]. Subsequent years were equally as warm, with some even warmer; The WMO [35] recognised 2016, 2019, and 2020 as the warmest years on record [35]. Since 2000, Europe has been affected by severe droughts, particularly in: 2003, 2006, 2010, 2015, 2018, 2019 and 2020 [36,37].

Recent years have been exceptionally warm and dry also in Poland. According to the Meteorological Yearbook [38], 2019 with the annual mean air temperature of 10.2 °C (this temperature was higher by 2.4 °C than the multiannual mean value from the period between 1971 and 2000) was the warmest year in the last 50-year period in Poland. The following year, despite being cooler than 2019, was overall one of the warmest years in the last 50-year period. The annual mean air temperature in Poland in 2020 was 9.9 °C and was higher than the normal multiannual value from the period between 1981 and 2010 by 1.7 °C [38]. Many parts of the country have suffered from drought in recent years.

Additionally, in the region of the Polish Carpathians in the last decades, the threat of meteorological drought has increased. The main cause is the increased air temperature as no significant decrease in precipitation has been observed in a multiannual period, whereas precipitation is characteristically highly variable from year to year [39].

The main aim of this article was to establish the spatial variability of the annual maximum drought in the region of the Polish Carpathians and the risk of maximum drought of a duration and volume exceeding the given value occurring in this area.

The present study constitutes a continuation of articles [40,41], however the findings of the previous analyses are not directly used herein, mainly due to the fact that the aforementioned works were based on flow series from different (earlier) multiannual periods. Baran-Gurgul [40] contains comprehensive information on droughts in the rivers of the Polish Carpathians. The article also includes an analysis of the spatial variability of the basic drought characteristics, the seasonality of the start- and end-time of droughts, and the number of drought days, as well as the multiannual variability of the number of drought days. The latter of the two articles, Baran-Gurgul [41], sought to assess whether the gamma distribution may be used to describe the distribution of the duration and volume of annual maximum drought in the Upper Vistula Basin.

This was conducted based on a 30-year time series of daily flows in selected gauging cross sections on rivers in the Polish Carpathians.

The scope of the study included estimating drought series, establishing their basic characteristics (the duration and volume), and based on the findings, estimating the annual maximum drought series. Furthermore, each of the series served to construct a probability distribution of the annual maximum drought duration *T_max_*, annual maximum drought volume *V_max_*, and finally, the joint distribution of these variables. The obtained distributions allow for the creation of maps with the spatial distribution of maximum drought of a given return period. These types of maps provide information on the scale of maximum drought hazard in a given cross section (or area).

Hydrological drought may be described using a multitude of characteristics which may be strongly related. Due to the fact that the duration and volume of droughts are highly correlated variables, a more effective, bivariate approach was used to describe drought, which consists in estimating the joint probability of drought characteristics. This way, it was possible to eliminate the problem connected with the traditional univariate probability analysis, which may lead to over or underestimation of the involved hydrological risk. The most disastrous droughts for society and the economy are the ones longest in duration and highest in volume, therefore the authors discussed the annual maximum droughts. The methodology proposed in the article, namely an advanced approach based on bivariate copulas, may be utilized in monitoring the characteristics of maximum drought probability based on continuously updated flow sequences.

Against this background [42] work stands out, its aim was to compare the multi-year time series of the SPI in the reference period between 1984 and 2018 with those of the near future: the period between 2018 and 2050 (from a regional climate model) over Ankara Province, Turkey (in five meteorological stations). This splitting of the data string is an interesting approach which I am keen to use in my future research. Asfhar et al. [42] concluded that droughts (including extreme droughts) in Ankara would become more severe.

Data from as many as 40 gauging stations located in the area of the Polish Carpathians were used for the validation of the proposed approach in my work methodology. Thanks to the use of a large pool of data, I can estimate when extreme low flows occur most often in the Carpathian region, or in which part of the studied area they are longer and of greater volume. This information, including both the duration and volume of the low flow, determined for the mountain area of Poland, is, in my opinion, important in planning the risk of drought in this area.

To sum up, the proposed method ensures a comprehensive approach to estimating hydrological drought within a studied area and allows for an assessment and analysis of the maximum drought frequency, especially in a regional setup and can serve as a useful tool for natural resources management, especially in mountainous regions.

## 2. Materials

**Figure 1:**
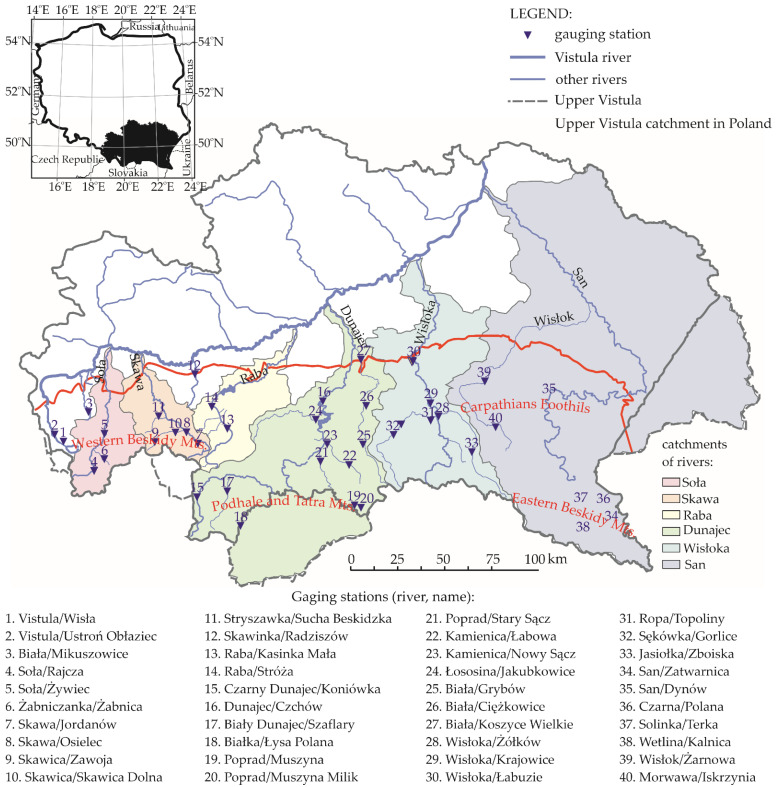
Study area and location of water gauging stations.

The area selected for this study includes the Carpathian part of the Upper Vistula basin (Figure 1). The Polish Carpathians cover 19,600 km^2^ which makes up approximately 6% of the Polish surface area [43]. A total of 87% of the Polish Carpathians territory belongs to the Western Carpathians, which include the Outer and Central Carpathians, while the remainder is the Eastern Carpathians. The Inner Carpathians include the Tatras and Podhale, which were formed by folding in the Late Cretaceous period, whereas the Outer Carpathians include the Beskidy and Carpathian Foothills, which were formed by folding in the Late Paleogene and Miocene period. Lying on the border of two mountain ranges, is the Pieniny Klippen Belt, which was folded in both early and late epochs. The highest range of the Polish mountains, as well as of the Carpathians, are the Tatras, and their highest peak is Rysy (2499 m a.s.l.). A detailed description of the region with the official subdivision into physiographic regions were included in the journal article by Baran-Gurgul [41].

There are 40 cross sections within the researched area. The data used for the calculations was obtained from the Institute of Meteorology and Water Management–National Research Institute (IMWM-NRI) as daily discharge series from the period between 1 November 1991 and 21 October 2020 (30 hydrological years which, in Poland, runs between 1 November and 31 October) in these cross sections (Figure 1, Table 1). Each series included 10,958 daily flows. Data are available at: https://danepubliczne.imgw.pl accessed on 30 July 2020.

**Figure 2:**
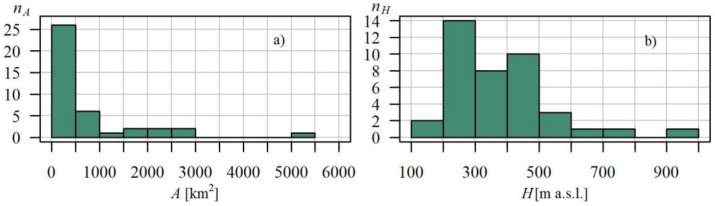
Plots show: (**a**) number of catchments versus catchment area *A*; (**b**) number of catchments versus gauging station elevation *H*; scales, in Polish Carpathian.

Gauging cross sections enclosed basins of surface areas ranging between 23.9 km^2^ and 5317.3 km^2^, whereas the gauge elevation was between 184.7 m a.s.l. and 965.6 m a.s.l. The arrangement as well as the relationship between basin area *A* and the gauging station elevation *H* is presented in Figure 2.

Half of the gauging station elevation values did not exceed 300 m a.s.l. (Figure 2b), and these were the gauges located within the region of the Beskidy Foothills and the northern part of the Beskidy Mts. region. The remaining gauges were located somewhat higher within the remaining part of the Beskidy Mts. and Bieszczady. Cross sections on the Upper Dunajec (within the Podhale and the Tatra Mts. region) were located at altitudes over 600 m a.s.l., with the highest-located cross section of Łysa Polana (17) on the Białka (965.6 m a.s.l).

**Figure 3:**
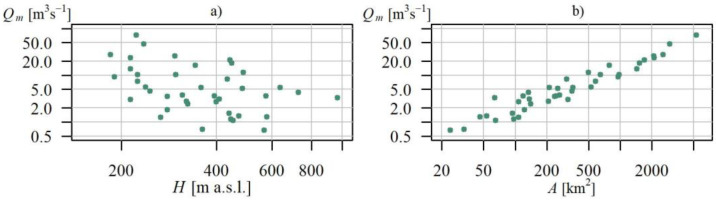
Scattergraph of relationship between average flow *Q_m_* and: (**a**) catchment area *A*; and (**b**) gauging station elevation *H*, in Polish Carpathian, in the logarithmic (log-log) scales.

Nearly all the basins (43 out of 44) had surface areas below 3000 km^2^, and half of the basins had areas below 300 km^2^ (Figure 2a). The largest basin enclosed by the Czchów cross section (16) on the Lower Dunajec had an area of over 5000 km^2^. Due to the land topography, the largest basins within the studied area were located in the northern part of the Carpathians with the mean flow *Q_m_* there often being higher than in the remaining area (Figure 3a).

As could be expected, mean flow is highly correlated with the basin area; in the linear scale, this coefficient is 98%, while the coefficient of logarithmic values correlation is 96% (Figure 3b).

## 3. Methods

### 3.1. Streamflow Drought Definition

Streamflow drought is most commonly defined as a continuous period during which streamflow at a given cross section is below the assumed threshold value of the *Q_g_* flow. This definition of droughts, known as the threshold level method, was introduced by Yevjevich [44] in the US, and by Zielińska [17] in Poland. In order to calculate a drought, one of three methods is usually applied: the POT (Peak Over Threshold); the MA (Moving Average); and the SPA (Sequent Peak Algorithm) [45].

Assuming that *Q_g_* = *Q_p_*_%_ equates to assuming that a drought flow will be exceeded on average *p*% times a year, for example flow *Q*_90%_ is the flow value reached or exceeded during the 70% of the observation time in a multiannual period, which is, on average, 256 days a year. Tallaksen and van Lanen (2004) recommend that threshold flows valued between *Q*_70%_ and *Q*_95%_ be applied to calculating droughts on permanent rivers. The most often applied flows are *Q*_70%_ and *Q*_90%_ [1,14,15,45,46,47,48,49]. Methodology [50] widely recommended in Poland considers three threshold flows (*Q*_70%_, *Q*_90%_, and *Q*_95%_), wherein a drought defined by the *Q*_70%_ flow is called an ordinary drought and denotes a warning in a river, at *Q*_90%_–it is called a deep drought (defines an emergency state), while at 95% it is an extreme drought (which corresponds to a natural disaster).

In the present article, a drought is defined by the POT method (Peak Over Threshold, N.B. a misleading name originating from the analyses of maximum flows). According to this method, a drought is a period of time *T* during which streamflow is below the flow *Q_g_*, therefore the start of a drought tp occurs when the flow falls below the *Q_g_*, and the end of the drought is when river streamflow rises back to the *Q_g_* level and exceeds it [51]. *Q*_70%_ was assumed as the threshold level of *Q_g_*.

In order to establish the primary drought characteristics based on the multiannual daily discharge series *Q_t_* at a given gauging cross section, time series of the drought start *t_beg_*_,*i*_ and the drought end *t_ene_*_,*i*_ (*i* = 1, 2 …) were created, thus helping define different drought characteristics:

-duration *T_i_* of the *i*-th drought (in days):

(1) $$T_{i}=t_{end,i}-t_{beg,i}+1\;$$

-drought volume *V_i_* (in days):

(2) $$V_{i}=\sum_{i=t_{beg,i}}^{t_{end,i}}\left(Q_{g}-Q_{i}\right)\;/\bar{Q}\;$$

where $\bar{Q}$ is a mean daily discharge from a multiannual period.

Measuring the drought volume in days (2) shows the number of days needed to supply a drought with a mean discharge at a given cross section, which allows to compare the volumes of droughts at various gauging cross sections. This approach has been used previously in the author’s work [41].

A number of authors have applied additional assumptions for defining a drought, e.g., its minimum duration or the inter-event time criterion, which involves combining two adjacent droughts when the interval separating them is shorter than the given value. Similar to article [41], droughts were estimated with the primary POT method, without the use of additional conditions (filters).

### 3.2. Maximum Drought Definition

Maximum droughts are known as maximum [13,14] or extreme [1,15]. This definition may relate to a maximum drought in a given period of time, in general a year or part of the year, or a whole multiannual period. According to the extreme value theory, a maximum drought may be defined in two ways: using the AMS (annual maximum series) model or the PDS (partial duration series) model. The first one consists in selecting the annual maximum drought duration (annual maximum volume) in a given multiannual period and identifying the probability distribution of this characteristic. The second one includes the selection of all the drought durations (volumes) over the assumed threshold value and combined probability analysis of the number and magnitude of exceedance of this value in a year [4]. The PDS practically equates to the AMS for the probabilities of exceedance below 10%, therefore for these probabilities the less complex approach of the AMS may be used successfully.

In this paper, two definitions of annual maximum drought (further called maximum) were assumed:

- the drought of the longest annual duration *T_max_*;
- the drought of the largest annual volume *V_max_*.

If a maximum drought starts in one hydrological year and ends in the following one (a drought moving from one year to the next), it is not divided but included as a whole in the year in which its middle had occurred. This approach was proposed by [15].

### 3.3. Stationarity of Time Series T_max_ and V_max_

The basis of the method utilised in this study for analysing the frequency of extreme events (e.g., maximum droughts) was the assumption of the stationarity of the random sample [4,52,53,54]. This condition means that the environmental mechanism of generating the duration and volume of maximum droughts is the same in each year (drought characteristics are from the same probability distribution) and that the past values of a variable do not affect the future values (lack of trend, time independence). Such an approach assumes that the hydrometeorological processes which bring about a drought occur in a little-changing natural environment, meaning that climate change or, for example, basin covering do not affect a drought [4].

The most popular test for assessing the homogeneity of drought characteristics, recommended by, among others, the WMO (2009) is the Mann–Kendall test [55,56,57]. In the paper, the Mann–Kendall test with the Hamed and Rao correction for autocorrelation was used for assessing the stationarity of the time series of duration *T_max_* and volume *V_max_* of annual maximum droughts.

Given was the time series *X_i_* (*X_i_* = *T_max_*_,*i*_ or *X_i_* = *V_max_*_,*i*_), *i* = 1, 2, …, *n*. The Mann–Kendall test [58,59,60] verified the hypothesis H_0_ of the homogeneity of the time series *X_i_* (more specifically, variables *X_i_*, *i* = 1, 2, …, *n* were independent and had identical distributions), with the alternative hypothesis H_1_ of the presence of a monotonic trend.

The condition for applying the Mann–Kendall test is the lack of autocorrelation. Positive autocorrelation increases the probability of type I error, namely detecting a significant trend despite one not being present [61]. Negative autocorrelation has the opposite effect, var(S) is overestimated and the test is unable to detect the present trend, which means that type II error will have occurred [62]. Hydrological time series, such as discharge time series, show positive autocorrelation [61]. In a situation where the autocorrelation is significant, Bayley and Hammerslay [61] proposed a variance correction with the use of the so-called effective number of observations.

### 3.4. Probability Distributions of the T_max_, V_max_ Series

In this paper, for the analysis of maximum droughts, the AMS method was applied, which consists in determining maximum characteristics in a year. Due to the fact that droughts do not always occur in each year, the maximum time series in the year of duration and volume of maximum drought may include zero values. In this case, distribution *F*(*x*) of the duration or volume of a maximum drought becomes a mixed distribution, more specifically discrete-continuous [63]:

(3) $$F(x)=p_{0}+(1-p_{0})G(x)$$

where *p*_0_ = P(*X* = 0), a *G*(*x*) = P(*X* > *x*|*X* > 0) is continuous distribution function of non-zero values of the variable *X* (*X* = *T_max_*, *V_max_*).

Because one of the aims of this study was to obtain information relating to an area on possible maximum droughts, finding the best probability distribution for the characteristics of these droughts was performed in two stages. First, the best distribution in a given gauging cross section was selected, and then optimal distribution was determined for the whole area.

In the first stage, in each of the gauging cross sections, probability distributions of maximum drought duration and volume were identified with parameters estimated using the method of L-moments. Distribution fit to data was assessed using the Anderson–Darling test.

Identifying the probability distributions of duration and volume of maximum droughts consists in selecting the best probability distribution from among the chosen set of distributions. In the literature, the most common distributions used for defining these characteristics are bi- and trivariate [4,13,46]. In the present study, bivariate distributions were selected (defined by the parameters of scale and shape) for three main reasons. First, due to the limitations of the design of statistical models (including the hydrological ones), it is recommended to sparingly apply the number of distribution parameters [64,65]. Second, due to the small number of parameters, this approach was the simplest one, which may also affect the clarity of the obtained results. Third, the systematic and mean squared errors of the estimation of large quantiles may be smaller in case of bivariate distributions compared to their trivariate counterparts, especially for smaller samples of the count less than 50 [65,66].

The set of bivariate probability distributions of duration and volume of maximum drought chosen in the analysis included five distributions: normal, lognormal, Weibull, and gamma.

The method of L-moments was chosen for estimating the parameters of probability distribution. The method was selected as the best one in the study by Baran-Gurgul [40].

In the present paper, for the assessment of distribution fit, the Anderson–Darling test was used. Test statistics of this test is the following [67]:

(4) $$AD^{2}=-n-\frac{1}{n}\sum_{i=1}^{n}\left(2i-1\right)\left[\ln F(X_{\left(i\right)})+\ln(1-F(X_{\left(n-i+1\right)})\right]$$

where *F*(⋅) is the cumulative distribution function of the tested distribution, whereas *X*_(*i*)_, *i* = 1, 2, …, *n*, is the *i*-th value of the random sample (i.e., duration of volume of the maximum drought) in ascending order.

If in a given gauging cross section the goodness of fit test did not reject more than one distribution, it is necessary to choose the best one. The most popular criteria of choice are the Akaike information criterion (AIC) and the Bayesian information criterion (BIC). These criteria may not be applied if the parameters of distributions are estimated using a different method than that of the highest reliability, therefore Jakubowski [14] proposes selecting a distribution for which the *p_v_* value of the χ^2^ test is the greatest. In this paper, the best distribution in a given cross section was the one for which the *p_v_* value of the Anderson–Darling test was the greatest.

After selecting the best probability distributions of duration *T_max_* and volume *V_max_* of the maximum drought in particular gauging cross sections, it may often turn out that different distributions were chosen as the best ones in different gauging cross sections. In such a case, considering the comparability of results, it is necessary to choose one distribution. Therefore, in the second stage of the distribution identification, a distribution which was optimal for the studied area was chosen. It was assumed that the optimal distribution would be the one which was the best most often according to the Anderson–Darling test in the gauging cross sections within this area, while in the remaining cross sections, the distribution was approved by the Anderson–Darling test at the assumed significance level (α = 5%).

### 3.5. Joint Distribution of (T_max_,V_max_)

The most important maximum drought characteristics are the duration and volume. These variables are highly correlated. Therefore, the issue of the joint distribution of these variables seems interesting. Two approaches are commonly used in such a case. The first one, traditional, involves assuming a bivariate distribution of variables (*T_max_*,*V_max_*), often lognormal [14,18]. A limitation to this approach may be the fact that the marginal distributions are then the same (with accuracy to the parameters, of course), which may not be the best approach. This is why, for some time now, the applied approach has been the copula which enables constructing a joint distribution of many variables with different distributions.

Not always is the longest drought in a year is also the one of the highest volume, while the one of the highest volume is usually the longest one in a year. As the differences between $T_{max}^{g}$ and $T(V_{max}^{g})$ (and $V_{max}^{g}$ and $V(T_{max}^{g})$) are, in most cases, very small, therefore in the present paper, the joint distribution of duration $T_{max}^{g}$ and volume $V_{max}^{g}$ of a maximum drought will be analysed.

Copula-based multivariate distribution was proposed in 1959 [68]. Using the copula function, two marginal distributions were combined into a joint distribution. Bivariate copula (or bivariate cumulative distribution function) is function *C*: [0, 1]^2^ → [0, 1], which meets the following conditions [69]:

1. for each pair (*t*, *v*) ∈ [0, 1] the following is inferred:

(5) C(t,0)=C(0,v)=0 i C(t,1)=t, C(t,v)=v

copula is nondecreasing in both of its arguments, i.e., for *t*_1_ ≤ *t*_2_, *v*_1_ ≤ *v*_2_:

(6) $$C(t_{2},v_{2})-C(t_{1},\;v_{2})\geq C(t_{2},\;v_{1})-C(t_{1},v_{1})\;$$

The most important proposition within the copula theory is Sklar’s theorem which explains the role a copula plays in the correlation between multivariate cumulative distribution functions and univariate marginal cumulative distribution functions [69]. In the bivariate approach, this theorem states that if a continuous random variable (*X*,*Y*) has a joint cumulative distribution function *F_XY_* and the marginal distribution functions *F_X_* and *F_Y_*, then there is exactly one copula *C*: [0, 1]^2^ → [0, 1], such as:

(7) $$F_{XY}\left(x,y\right)=C\left(F_{x}\left(x\right),F_{Y}\left(y\right)\right)$$

An inverse is also true, meaning that if *C* is a copula and *F_X_* and *F_Y_* are the cumulative distribution functions of variables *X* and *Y*, then function *F_XY_* defined according to (7) is a joint cumulative distribution function of variable (*X*,*Y*) with marginal cumulative distribution functions *F_X_* and *F_Y_*.

For the analyses, four widely used [19,20,27,70] univariate copulas of variable $\left(T_{max}^{g},V_{max}^{g}\right)$ were selected, namely Plackett copula and Archimedean copulas (Clayton, Frank and Gumbel–Hougaard) (Table 2) with gamma distribution as marginal distribution of both duration $T_{max}^{g}$ and volume $V_{max}^{g}$ of maximum drought.

Similarly to the univariate perspective, in which for defining the duration and volume of maximum drought in gauging cross section *g* the best distribution was sought from among the chosen set of distributions, also the identification of joint distributions of variables $T_{max}^{g}$ and $V_{max}^{g}$ consisted in selecting the best probability distribution of variable $\left(T_{max}^{g},V_{max}^{g}\right)$, constructed based on one from the set of the proposed copulas.

The classical method of reliability of estimating copula parameters, in case of a bivariate model may be complex as it requires simultaneous estimation of the marginal distribution parameters and the copula parameters. This is why the inference function for the margins (IFM) is most commonly used in practice. This method, introduced by [71], is applied in two stages [72]. In the first stage of the procedure, the parameters of marginal distributions were estimated using the method of highest credibility. In the second stage, based on the set of estimated distribution parameters, parameters responsible for the correlation of variables, represented by a copula, were estimated. The method of highest reliability is most often used to estimating the parameters of copulas (including the duration and volume of droughts) [19,23]. Works can be found, however, where authors estimate unknown copula parameters in other ways, e.g., Tosunoglu and Kisi [30] used this to extend the method of moments.

In the literature, the widely applied goodness of fit tests for joint distributions are: the Cramer-von Mises test [24,30], χ^2^ [14] test, or the RMSE criterion [23,26,73]. Genest et al. [74,75] recommend the use of the Cramer-von Mises or Anderson–Darling tests.

In the present paper, the procedure of identifying the joint distribution of variables (*T_max_*, *V_max_*) was similar to identifying univariate distribution of variables *T_max_* and *V_max_*. First, the best distribution in a given gauging cross section was selected, and then the best distribution in the studied area. And in the same way as in the univariate case, the goodness of fit of the distribution to data was determined using the Anderson–Darling test, this time, however, its bivariate version (Genest et al., 2013) [75]. The best distribution in the given gauging cross section was selected as the one for which the *p*-value of the Anderson–Darling test was the greatest, calculated using the Copula package of the GNU R (R Core Team) software, version 4.2.1 [76].

### 3.6. Joint Return Period of Duration and Volume of Annual Maximum Drought

Return periods of each variable *T_max_* and *V_max_* analysed separately provide information on each individual variable. Therefore, the value of the return period of variable *T_max_* was calculated for all of the values of *V_max_* and vice versa, and the obtained result was, in a way, over- or underestimated. This is why a return period which concerns both variables *T_max_* and *V_max_* at the same time is more precise.

In case of a pair of variables (*X*,*Y*), joint bivariate return period *T_P_*(*x*,*y*) of event (*X* ≥ *x*, *Y* ≥ *y*) is defined analogously to the univariate case as an inverse of the distribution of this event:

(8) $$T_{P}(x,y)=\frac{1}{P(X\geq x,Y\geq y)}$$

which, in the event of variable (P(*X* ≥ *x*, *Y* ≥ *y*) = P(*X* ≥ *x*)P(*Y* ≥ *y*)) independence leads to a simple formula:

(9) $$T_{P}(x,y)=T_{P,X}(x)\cdot T_{P,Y}(y)$$

When the pair of variables is correlated, taking this fact into consideration leads to a formula for joint exceedance probability:

(10) $$P(X\geq x,Y\geq y)=1-F_{X}(x)-F_{Y}(y)+F_{XY}(x,y)$$

which, finally, offers a more complex formula for joint return period:

(11) $$T_{P}(x,y)=\frac{1}{P(X\geq x,Y\geq y)}=\frac{1}{1-F_{X}(x)-F_{Y}(y)+F_{XY}(x,y)}$$

If copula *C*[.] is applied, the above formula becomes:

(12) $$T_{P}(x,y)=\frac{1}{1-F_{X}(x)-F_{Y}(y)+C\left[F_{X}(x),F_{Y}(y)\right]}$$

Sometimes, the joint return period of the $\left(T_{max}^{g},V_{max}^{g}\right)$ value is defined differently, as an inverse of the probability of the exceedance alternative, not an inverse of the probability of the exceedance conjunction:

(13) $$T_{P}^{or}(x,y)=\frac{1}{P(X\geq x\mathrm{lub}Y\geq y)}=\frac{1}{1-C\left[F_{X}(x),F_{Y}(y)\right]}$$

This approach was not applied in this study.

Due to the fact that droughts do not always occur in each year of the studied multiannual period, the maximum time series from the year of drought duration and volume may include zero values. Then, as mentioned before (see Section 3.4), the distribution of duration *T_max_* or volume *V_max_* of a maximum drought, together with the bivariate distribution (*T_max_*, *V_max_*) becomes a mixed distribution, discrete-continuous. In such a case, Formula (13) is:

(14) $$T_{P}(x,y)=\frac{1}{P(X\geq x,Y\geq y)}=\frac{1}{1-F_{X}(x)-F_{Y}(y)+F_{XY}(x,y)}$$

where *p*_0_ = P(*X* = 0, *Y* = 0) is the probability of a year without a drought occurring in the studied 30-year period.

## 4. Results and Discussion

### 4.1. Primary Drought Characteristics

Drought does not occur in all of the years. In 34 cross sections, all of the years were with droughts. More than one year without a drought was observed in the following cross sections: Mikuszowice on the Biała river (three years) and Radziszów on the Skawinka river (two years).

**Figure 4:**
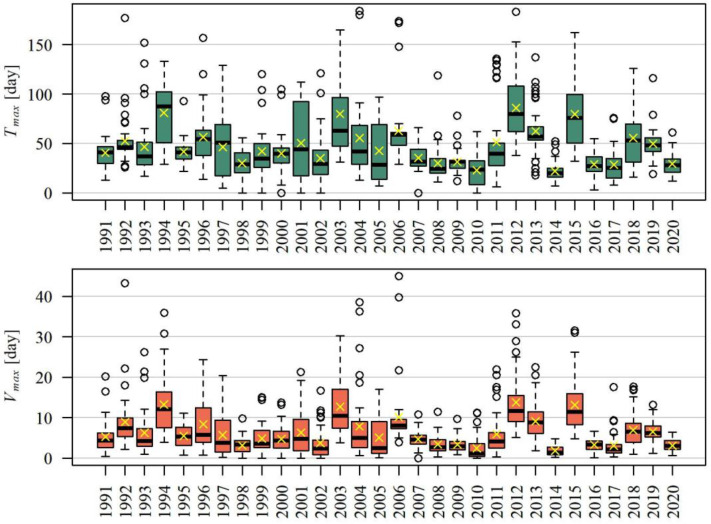
Distribution of the duration *T_max_* and volume *V_max_* of the maximum drought in the hydrological period between 1991 and 2020, in the studied area.

Primary extreme drought characteristics such as duration *T_max_* and its volume *V_max_* showed variability in the multiannual period (Figure 4). The longest droughts and droughts of the highest volume were observed primarily in 1987, 1994, 2003, 2012, and 2015–these were the years of the great droughts, often stretching across all of Europe.

**Figure 5:**
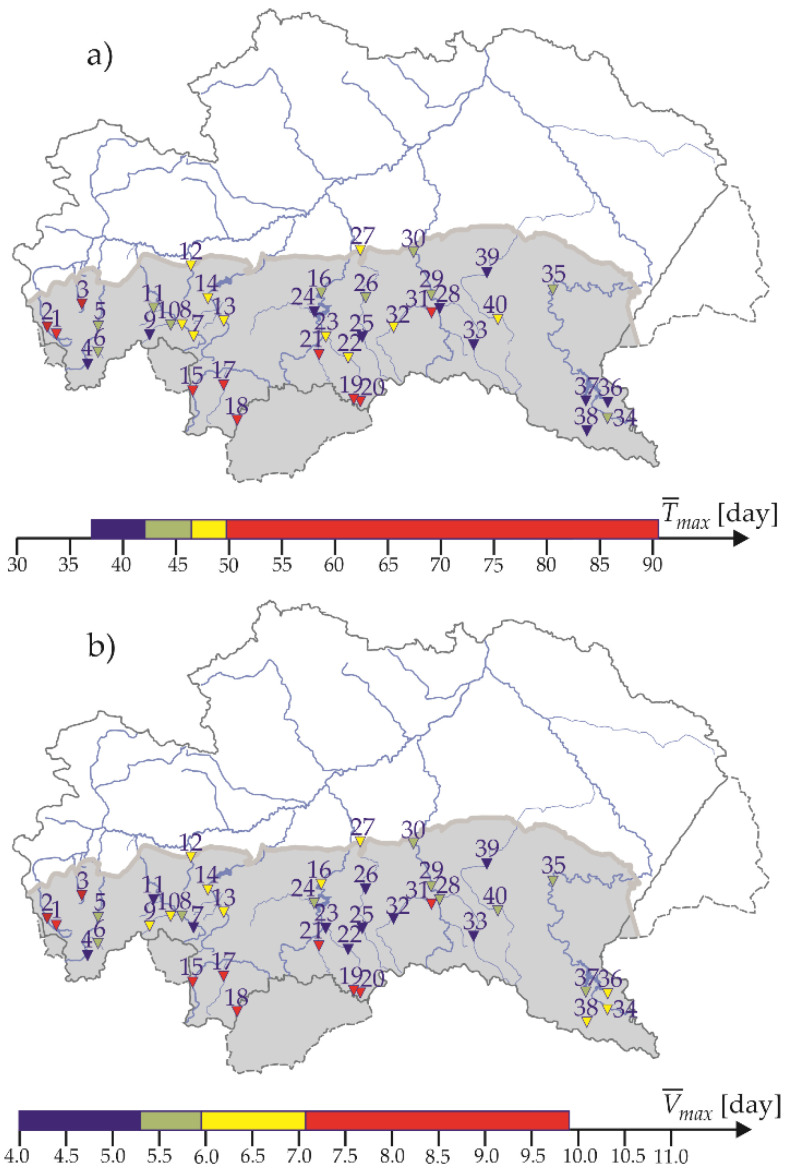
Spatial distribution of: (**a**) mean drought duration *T_max_*; and (**b**) mean drought volume *V_max_*, according to the quartile classification, including the box and whiskers plots in the hydrological period between 1991 and 2020.

Annual maximum drought duration in the period between 1991 and 2020 in the studied area was, on average, 47.3 days (median is 41 days). The longest mean drought *T_max_* and drought *V_max_* of the largest mean volume occurred in the Tatras in the Podhale, in the Little Vistula basin (in the western part of the studied area) as well as the southern, the highest part of the Dunajec river basin (Figure 5). In her research, Tlałka [77] reached completely different conclusions, and observed that droughts in the Tatras and Podhale region are short. The discrepancy of results may result from the fact that Tlałka considered the summer droughts only, while long high-volume droughts in this area occur during autumn and winter.

The lowest values ${\bar{T}}_{max}$ and ${\bar{V}}_{max}$ were observed in the central, the Beskidy part of the Upper Vistula basin. A note-worthy situation takes place in the Bieszczady, where values ${\bar{T}}_{max}$ were the lowest in the studied area, however these droughts were not of the lowest mean volume *V_max_*. Tlałka [77] found that droughts in the Bieszczady and the Beskidy Mountains are short, whereas their durations vary in the Beskidy Foothills area.

The longest observed droughts lasted 184 days (between 4 August 2003 and 3 February 2004) occurred on the Poprad river, at the Muszyna Milik and the Stary Sącz cross sections. Droughts were assigned by their middle; therefore these ones were included in the year 2004.

The volume of the maximum drought in the period between 1991 and 2020 was, on average, 6.4 days (the median is 4.8 days). The drought of the highest volume (*V_max_* = 45 days) occurred between 8 August 2003 and 2 February 2004 (174 days long) at the Czchów cross section at the Dunajec river.

Duration *T_max_* is highly correlated with *V_max_*, depending on the gauging cross section, the correlation coefficient assumed values between 83% and 97%.

**Figure 6:**
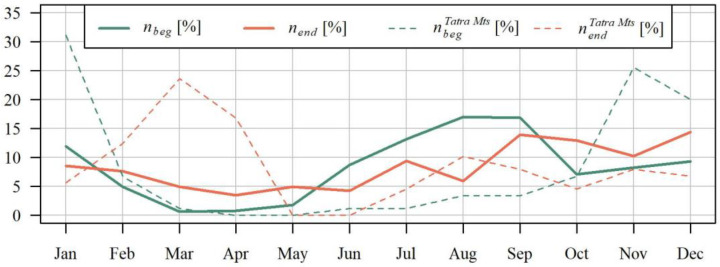
Distribution in the year of the mean relative number of the maximum drought beginning *t_beg_* and end *t_end_* in the studied area and $t_{beg}^{TatraMts},t_{end}^{TatraMts}$ in Tatra Mts., in the hydrological period between 1991 and 2020.

Extreme droughts in the Polish Carpathians region start most often at the turn of summer and autumn (mainly in August or September), and end most often in autumn (mainly in August, September, or October) (Figure 6). Against these, maximum droughts observed in the Tatras in the Podhale stand out: they are hibernal, and usually start in November and end in March.

Ziemońska [78] reached similar conclusions. While studying water relations in the Western Carpathians, she observed that the minimum flows in the Tatras, the Silesian and the Żywiecki Beskid occur in winter, whereas in the remaining areas the minimum flows occur in autumn. Fal (2007) [79] observed that it is most often in mountain rivers that winter droughts occur, whereas summer droughts are often shorter compared to areas of lower elevation, e.g., due to high permeability of the typically mountainous rocky soil.

### 4.2. Stationarity of Duration and Maximum Drought Volume Time Series

**Figure 7:**
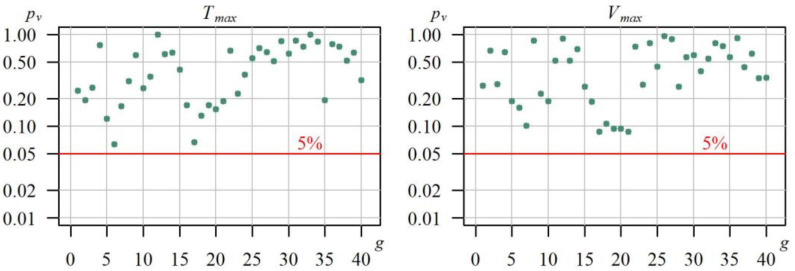
The p_v_-value obtained from the Mann–Kendall test for the time series of duration $T_{max}^{g}$ and volume $V_{max}^{g}$ of the maximum drought in the hydrological period between 1991 and 2020.

All the time series of duration $T_{max}^{g}$ and volume $V_{max}^{g}$ of annual maximum drought in each of the 40 gauging cross sections were subjected to the Mann–Kendall test with a possible margin for autocorrelation. In none of the analysed cases, at the significance level of 5% (*p_v_* < 5%), was there any basis for rejecting the hypothesis on the lack of the time series trend $T_{max}^{g}$ and $V_{max}^{g}$. The *p_v_* values of the hypothesis test in all the analysed cases are shown in Figure 7.

### 4.3. Probability Distribution of Duration and Probability Distribution of Volume of a Maximum Drought

**Figure 8:**
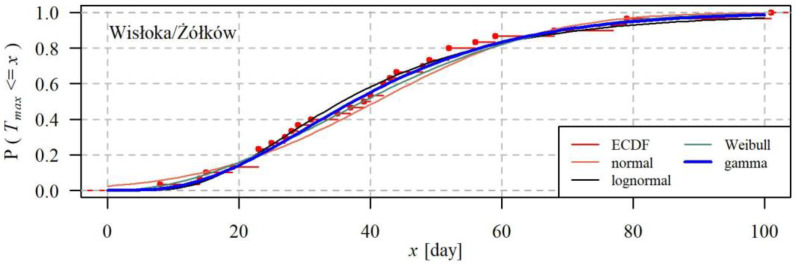
Empirical cumulative distribution ECDF and the normal, lognormal, gamma and Weibull probability distributions of variable *T_max_* at the Żółków gauging cross-section of the Wisłoka river.

In each of the 40 gauging cross sections, assuming that the time series of the *T_max_* duration and the *V_max_* volume were stationary, the parameters of univariate probability distributions of the *T_max_* and *V_max_* variables were estimated using the method of L-moments. In the case of years without the occurrence of droughts, the applied distributions were mixed (discrete-continuous). Probability *p*_0_ of a year without a drought was estimated as a relative number of no-drought years in the studied 30-year period. The continuous part of the distribution was the best out of four bivariate probability distributions: normal, lognormal, Weibull, or gamma: Figure 8 shows the cumulative distribution functions: the empirical one as well as for the abovementioned distributions, as an example, for one of the stations.

**Figure 9:**
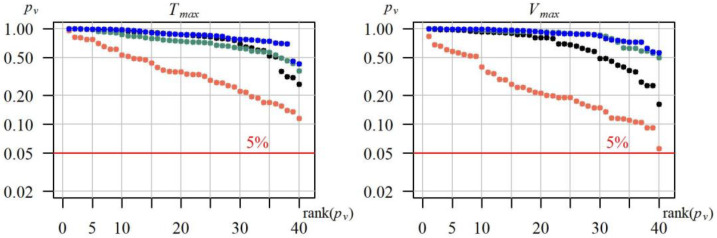
Distribution of descending *p_v_*-values of the Anderson–Darling goodness-of-fit test of four tested probability distributions (● normal, ● lognormal, ● Weibull, ● gamma) of the duriation *T_max_* and volume *V_max_* of the maximum drought, in 40 gauging cross-sections.

Distribution fit was assessed using the Anderson–Darling goodness of fit test. The best distribution in a given gauging station was determined based on the highest *p_v_*–value by the Anderson–Darling test. Figure 9 shows a comparison of the goodness of fit for the distributions of variables *T_max_* and *V_max_* from the assumed set of distributions at 40 gauging cross sections, expressed with values *p_v_* of the Anderson–Darling test.

In all the 40 studied gauging cross sections, probability distributions of the *T_max_* and *V_max_* characteristics of maximum droughts may be described at the level of significance of α = 0.05 using each of the tested distributions. As expected, due to the symmetry, the worst distribution for describing the drought characteristics was the normal distribution. The best distribution was the gamma, for which, in most cases, the *p_v_* values of the Anderson–Darling were higher compared to the remaining distributions.

**Figure 10:**
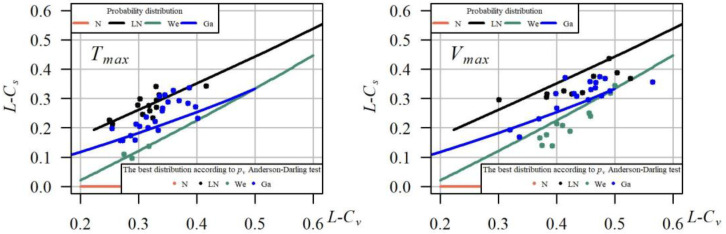
Dependence of the linear coefficient of skewness *L*-*C_s_* on the linear coefficient of variation *L*-*C_v_* for four tested probability distributions (N—normal, LN—lognormal, We—Weibull, Ga—gamma) of the duration *T_max_* and volume *V_max_* of the maximum droughts, in 40 cross-sections.

Another method for the qualitative assessment of the goodness of fit of distributions is a chart of theoretical relation between the linear skewness coefficient (*L-C_S_*) and the linear variation coefficient (*L-C_V_*) against the backdrop of a point cloud (*L-C_V_*,*L-C_S_*) of empirical values of these coefficients for a given random sample (Figure 10). The location of a point on the line corresponding with the given distribution or within its vicinity may indicate the best goodness of fit of this distribution to the data series. On most of the charts, the points are most often located in the vicinity of the lines corresponding with the gamma distribution, which strongly supports the previous suggestion that this distribution is best described by *T_max_* and *V_max_*.

To sum up the above considerations, it may be said that duration *T_max_* and volume *V_max_* of the maximum drought was best described by distribution gamma, therefore this distribution was chosen for further analyses.

### 4.4. Bivariate Distribution of Time Series of Duration and Volume of Maximum Droughts

The estimation of the copula parameter was performed using the maximum likelihood estimation, and the goodness of fit, similarly to the univariate perspective, was studied using the Anderson–Darling test (at the significance level of 0.05). Estimating both the parameters and the *p_v_* of the Anderson–Darling test was performed using the Copula package of the GNU R (R Core Team) software, version 4.2.1 [76].

**Figure 11:**
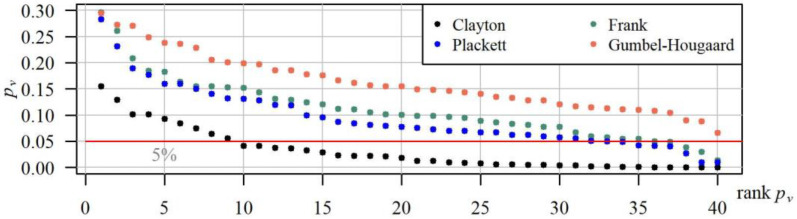
Distribution of descending *p_v_*-values of the Anderson–Darling goodness-of-fit test for distributions of the variable $\left(T_{max}^{g},V_{max}^{g}\right)$ based on copulas: Clayton, Frank, Gumbel–Hougaard and Plackett, in 40 gauging cross-sections.

In all of the 40 studied cases, the distribution of variable $\left(T_{max}^{g},V_{max}^{g}\right)$ may be constructed using the Gumbel–Hougaard copula (Figure 11).

Figure 11 shows the *p_v_* values of the bivariate Anderson–Darling test in descending order (individually for each copula). In general, the *p_v_* values estimated for the distribution constructed based on the Gumbel–Hougaard copula tend to be higher than the *p_v_* values for the distributions created using the remaining copulas. As a result, the joint distribution of duration and volume of a maximum drought based on this copula expressed the correlation between $T_{max}^{g}$ and $V_{max}^{g}$ more accurately than a distribution constructed with the other considered copulas. In 85% cases, the *p_v_* value of the Anderson–Darling test for the probability distribution created using this copula was higher than the *p_v_* for distributions created using Clayton, Frank, or Plackett copulas. Clearly, the worst turned out to be the Clayton copula, which may be accepted in merely 20% (in eight out of 40) of cases.

**Figure 12:**
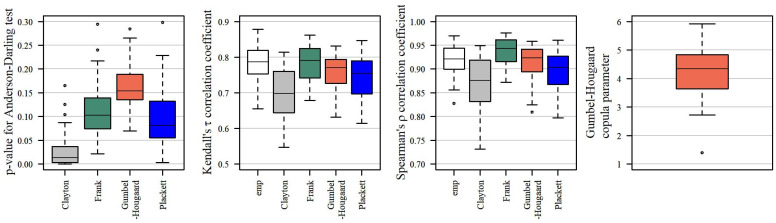
*p_v_*-values of of the bivariate Anderson–Darling goodness-of-fit test, Kendall and Spearman correlation coefficients of the variables $T_{max}^{g}$ and $V_{max}^{g}$: empirical and computed for the probability distribution by using copulas (Clayton, Frank, Gumbel–Hougaard and Plackett), and Gumbel–Hougaard copula parameter used to estimate the joint distribution of $\left(T_{max}^{g},V_{max}^{g}\right)$. The lower and upper borders of the box are the first and third quartiles and the line inside the box—the median value. The whiskers extend to 1.5× the interquartile range. The points outside the whiskers represent statistical outliers.

However, irrespective of the studied case or the type of applied copula, the correlation coefficients τ and ρ are statistically significant at the significance level of α = 0.05. Kendall’s τ correlation coefficients of variables $T_{max}^{g}$ and $V_{max}^{g}$ were lower than the Spearman ρ correlation coefficients (Figure 12). The lowest correlations, in the case of both coefficients, were observed in the case of variables generated from the probability distribution using the Clayton copula, and the highest using the Frank copula. Empirical values were closest to the coefficients of the correlation $T_{max}^{g}$ and $V_{max}^{g}$ generated from the probability distribution using the Gumbel–Hougaard copula.

Owing to the goodness of fit defined by the Anderson–Darling test, as well as the highest proximity of the theoretical Kendall and Spearman correlation coefficients to the empirical coefficients, the best joint probability distribution of variables $\left(T_{max}^{g},V_{max}^{g}\right)$ was the distribution constructed using the Gumbel–Hougaard copula with marginal gamma distributions. The values of the correlation coefficients τ and ρ generated from the probability distribution using the Gumbel–Hougaard copula, as well as the parameters of this copula, are summarised in Table 3.

**Figure 13:**
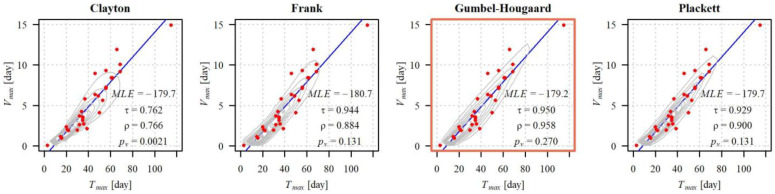
Empirical duration $T_{max}^{g}$ and volume $V_{max}^{g}$ of the maximum droughts (dots) and the density functions of the variable $\left(T_{max}^{g},V_{max}^{g}\right)$ distributions based on the given copulas in the Żywiec na Soła cross-section. The blue line represents the linear trend line.

For example, in Figure 13, there is a comparison of the density functions of probability distributions at all the applied copulas, at the gauging cross section Żywiec on the Soła river. Similar to most of the remaining cross sections, also in this case, the *p_v_* value for the probability distribution constructed based on the Gumbel–Hougaard copula was the greatest.

### 4.5. Joint Return Period of Duration and Volume of a Maximum Drought

**Figure 14:**
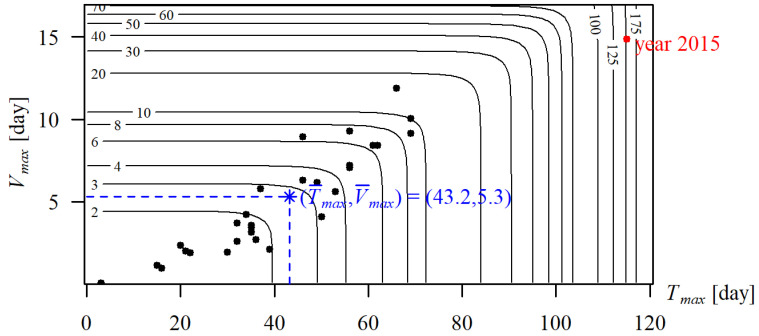
Contour plot for the joint return period *T_P_*(*T_max_*,*V_max_*) [in years] at the Żywiec cross-section of the Soła river. The black points indicate a random sample (*T_max_*,*V_max_*). * indicates *T_p_* for average values *(T_max_*,*V_max_*).

The return period calculated from the correlation (22) means that the same joint return period *T_P_* may be achieved for different values of random variables *X* and *Y*. Therefore, the joint return period *T_P_* of the duration $T_{max}^{g}$ and volume $V_{max}^{g}$ of a maximum drought in the 40 studied gauging cross sections was illustrated by means of an isoline (*x*,*y*) estimated using equation *T_P_*(*x*,*y*) = const, where each isoline matches a particular value *T_P_* being the return period of the duration of a maximum drought equal to or longer than the given value and the volume of a maximum drought equal to or greater than the given value. Owing to the large number of cross sections, Figure 14 shows exemplary distributions (isolines of the joint return period *T_P_*(*x*,*y*) of event (*T_max_* ≥ *x*, *V_max_* ≥ *y*)) of maximum droughts in cross section Żywiec on the Soła river.

This figure provides information on the values (*T_max_*,*V_max_*) for each *T_P_*, as well as joint return periods *T_P_* of historical droughts. For example, the greatest drought in the cross section Żywiec, in the studied period, occurred in 2015. It lasted 115 days and its volume was approximately 14.88 days. Such a drought, i.e., no shorter than 115 days and of a volume no lower than 14.88 days, occurred, on average, once every 150 years.

Maximum droughts of a given duration and volume, for example a drought no shorter than 43.2 days and of volume no lower than 5.3 days, occurs, on average, every three years.

Further analyses of the joint return period *T_P_*(*x*,*y*) of the duration and volume of a maximum drought were carried out assuming particular, determined for each gauge, values (*x*,*y*) = {(*T_max_*_,10_,*V_max_*_,10_), $\left({\bar{T}}_{max},{\bar{V}}_{max}\right)$)}, namely for 10-year and mean durations and volumes of maximum droughts.

**Figure 15:**
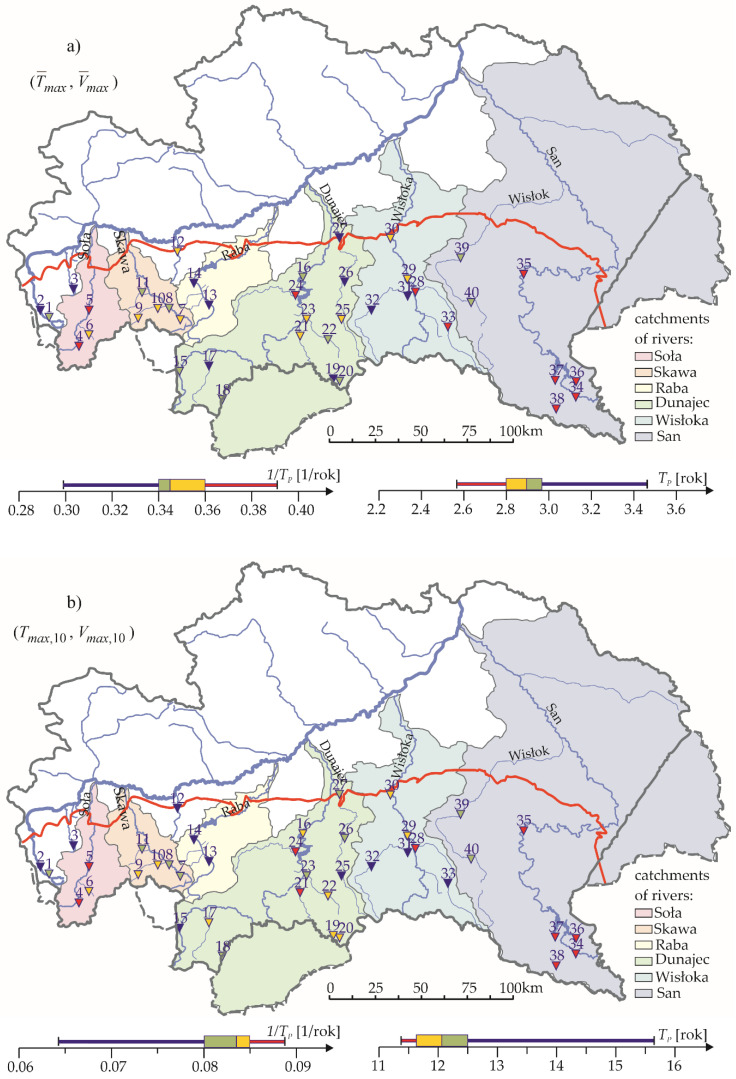
Spatial distribution of the frequency of the maximum drought: (**a**) $\left({\bar{T}}_{max},{\bar{V}}_{max}\right)$; and (**b**) (T_max,10_, V_max,10_), according to the quartile classification, including the box and whiskers plots 1/*T_p_* and *T_p_*, in the hydrological period between 1991 and 2020.

Spatial frequency distributions of the occurrence of droughts (*T_max_*_,10_, *V_max_*_,10_) and $\left({\bar{T}}_{max},{\bar{V}}_{max}\right)$ the values of particular quartiles 1/*T*P were summarised on the maps of the studied area, illustrating at the same time the scale of hazard of a *T_P_*-year maximum drought. A hazard is understood here as a value growing with the duration and volume of a maximum drought. In order to be able to compare these maps comfortably, the *T_P_* value ranges were divided into categories using the *T_P_* quartiles as threshold class values. Quantile classification enables qualitative comparison of the variability of different variables, especially when their value ranges differ significantly. The applied coding including matching colour key is presented in Table 4, and the maps of results–in Figure 15. The maps also include the values of particular quartiles.

These maps reveal more or less clear grouping of stations which belong to particular categories, as well as some basin-areal similarities or differences, as well as differences of occurrence frequency of the 1/*T_p_* maximum droughts (respectively 1/*T_P_*(*T_max_*_,10_,*V_max_*_,10_) 1/*T_P_$\left({\bar{T}}_{max},{\bar{V}}_{max}\right)$*). The spatial distributions of the frequency of drought occurrence (*x*,*y*) = {(*T_max_*_,10_,*V_max_*_,10_), $\left({\bar{T}}_{max},{\bar{V}}_{max}\right)$} shown in the maps were similar, and, therefore, will be described jointly.

The frequency 1/*T_P_* of maximum droughts (*x*,*y*) occurring in the area of the Little Vistula river basin (up to the estuary of the Biała river) was “the lowest” (and “moderate” in one of the cross sections), which means that the joint return period *T_P_* of a maximum drought of duration no shorter than *x* and volume no lower than *y* was “the longest” here (moderate in one of the cross sections) (Figure 15).

The frequency 1/*T_P_* of a maximum drought (*x*,*y*) occurring on the Soła river was included in “the highest” category, and on the Soła river tributary–the “high” category.

The grouping of the categories 1/*T_p_*(*x*,*y*) of maximum droughts POT-70% and SPA-70% in the area of the Skawa river basin was, depending on the cross section–“moderate” or “high”.

The frequency of maximum droughts occurring in the cross sections of the Raba river basin was “the lowest”.

The Dunajec river basin included three physico-geographical regions (the Subcarpathian, the Beskidy, and the Tatras-Podhale). The probability of maximum drought (*x*,*y*) occurring in the Subcarpathian (northern) and the Tatras-Podhale (southern) parts of the Dunajec basin was “low” and “moderate”, whereas in the Beskidy (middle) part of the Dunajec basin, the frequency of drought (*x*,*y*) occurring in the majority of the cross sections was most often “high”. The region of the Tatras and the Podhale is, however, at risk of long-lasting winter droughts.

The probability of droughts POT-90% and SPA-90% occurring in the Wisłoka river basin was varied, in the western part–the lowest, while in the eastern–high or the highest.

Clearly the highest values of 1/*T_P_* in the San river basin were observed in its upper part (the Bieszczady). In the remaining (the Beskidy) part of the San river basin, the probability of maximum droughts occurring was “moderate”.

To summarise, most of the cross sections in the Carpathian part of the Vistula basin, in which the probability of maximum droughts (*T_max_*_,10_, *V_max_*_,10_) and $\left({\bar{T}}_{max},{\bar{V}}_{max}\right)$ occurring was “the lowest”, occurred mostly in the basins of the Little Vistula, the Raba river, and the Wisłoka river. “The highest” drought hazard was observed mostly in the southern, Bieszczady part of the San river basin as well as the Soła river basin.

The results of the present study were similar to the conclusions from the “Drought Effects Counteracting Plan” project [80]. According to the Plan, the area most at risk of streamflow drought is the region of the Tatras and the Podhale (where long-lasting hibernal droughts occur), while the remaining part of the region of the right-bank part of the Upper Vistula basin was mostly considered being at great drought risk. The authors of the Plan agreed that the part of the Beskidy was at “moderate” risk of streamflow drought.

## 5. Final Remarks

This study concerned annual maximum droughts in the hydrological multiannual period between 1991 and 2020 in the gauging cross sections located in the Polish Carpathians. Annual maximum droughts here are understood in two ways: as the longest droughts in a year, or droughts of highest volume in a year.

Determined series of primary drought characteristics (duration and volume) were the basis for defining maximum droughts, which allowed for, among others, identifying the probability distributions of duration and volume, and, in consequence, defining the maximum droughts of a given return period (given risk level). Further analysis based on these calculations allowed for the selection of the areas more or less at risk of extreme annual drought of duration and/or volume exceeding the given value.

The longest maximum droughts, as well as those of the highest volume, were observed in the higher-located areas, the Little Vistula basin, and in the Tatras and the Podhale. Maximum droughts observed in the central part of the studied area (most prominently in the basins of the rivers: Soła, Raba, and Wisłoka), as well as in the south-eastern part of this area (in the Bieszczady, in the basin of the Upper San river) were most often shorter and had lower volume.

The longest droughts in a year in the region of the Polish Carpathians were the summer-autumnal ones, and the droughts in the Tatras and the Podhale were in winter, most often starting in November and ending in March.

The bivariate analysis of the frequency of characteristics of annual maximum droughts requires defining the stationarity of the series of these characteristics, and then defining the optimal marginal distributions of these characteristics. At the assumed significance level, there was no basis for rejecting the hypothesis of the lack of the time series trend $T_{max}^{g}$ and $V_{max}^{g}$. Because the studied characteristics in the series of maximum droughts were stationary, the best distribution of duration *T_max_* and volume *V_max_* was chosen out of the set of four distribution candidates. The best distribution (according to the Anderson–Darling goodness of fit test, at the significance level of 0.05) for the description of both characteristics of a maximum drought turned out to be the gamma distribution with parameters estimated using the method of L-moments.

The bivariate approach to studying droughts is a more wholesome form of analysis as it allows for the consideration of the duration and volume of a drought at the same time. For the description of the joint probability of duration *T_max_* and volume *V_max_* of a drought, the bivariate probability distribution constructed based on a copula function was used. Out of the four proposed copulas, the best one (according to the Anderson–Darling test) for the estimation of the probability distribution of variables (*T_max_*,*V_max_*) was the bivariate copula of extreme distributions, the Gumbel–Hougaard copula, with the gamma distribution as marginal distribution of both the duration *T_max_* and the volume *V_max_* of the maximum drought.

The return period *T_P_*(*x*,*y*) in the bivariate distribution is, in general, a function of variables *x* = *T_max_* and *y* = *V_max_*, hence some difficulty in presenting it graphically. This is why the joint return periods *T_P_*(*x*,*y*) were defined for the use of maps only for selected values (*x*,*y*), namely: *T_P_* (*T_max_*_,10_, *V_max_*_,10_) and *T_P_$\left({\bar{T}}_{max},{\bar{V}}_{max}\right)$*. Subsequently, based on the quartile classification, spatial distributions of frequency 1/*T_P_* of droughts (*T_max_*_,10_, *V_max_*_,10_) and $\left({\bar{T}}_{max},{\bar{V}}_{max}\right)$ occurrence were generated. In the description, four categories of 1/*T_P_* were assumed (the lowest, moderate, high, and the highest occurrence frequency).

In general, the return periods *T_P_* of drought occurrence (*T_max_*_,10_, *V_max_*_,10_) POT-70% exceeded, in the vast majority of the cross sections, 10 years by a few years. Therefore, it may be presumed that most often drought *T_max_*_,10_ was also drought *V_max_*_,10_.

Distributions *T_max_* and *V_max_* were right-skewed (asymmetrical), therefore the return (exceedance) period of the mean values was over two years. This is why a summary which would be analogous to the given for droughts (*T_max_*_,10_, *V_max_*_,10_) may be less precise for drought (${\bar{T}}_{max},{\bar{V}}_{max}$).

Spatial distributions of the frequency of drought occurrence 1/*T_P_* (*T_max_*_,10_, *V_max_*_,10_) and 1/*T_P_ $\left({\bar{T}}_{max},{\bar{V}}_{max}\right)$* of maximum droughts were not too dissimilar. The lowest probability of drought occurrence (*T_max_*_,10_, *V_max_*_,10_) and $\left({\bar{T}}_{max},{\bar{V}}_{max}\right)$ was mostly in the basin of the Little Vistula, as well as in the basins of the Raba river and the Wisłoka river. In the region of the Tatras, the frequency of drought occurrence was not high, however the droughts are long-lasting and winter.

In most of the analysed cases, the shortest (most often “low” and “moderate”) joint return periods *T_P_* (*x*,*y*) indicating the greatest chance of maximum drought of duration longer than *x* (equal to *T_max_*_,10_ or ${\bar{T}}_{max}$) and volume *y* (equal to *V_max_*_,10_ or ${\bar{V}}_{max}$, respectively) occurrence, were observed in the Bieszczady part of the San river basin, as well as the basins of the Soła river and the Skawa river.

## 6. Conclusions

On the basis of the analyses done on 30-year time series of daily discharges at 40 gauging cross sections located in the Polish Carpathians, the following conclusions may be drawn:

1. The longest maximum droughts and of the highest volume, occurred in the Little Vistula basin and in the Tatras in the Podhale;
2. Maximum droughts within the studied area were summer-autumnal, and in the Tatras or the Podhale in winter;
3. The gamma distribution may be used to define the duration *T_max_* and volume *V_max_* of the maximum drought in the region of the Polish Carpathians;
4. For the estimation of the joint distribution probability of variables (*T_max_*,*V_max_*), the Gumbel–Hougaard copula with the gamma distribution as marginal distribution of both the duration *T_max_* and the volume *V_max_* of the maximum drought may be used;
5. Within the Carpathian part of the Upper Vistula Basin, the areas with the highest drought risk are: in the summer-autumn season the basins of the Soła river, the Skawa river, or the Upper San river (the Bieszczady Mts.), whereas in winter–the Tatras and the Podhale (where the return period of droughts is not high, however droughts tend to be long and of high volume).

In Poland, where there is a small proportion of water resources per inhabitant, there is a large number of works and analyses being written on the subject of droughts, which are occurring more and more frequently. The “Drought Effects Counteracting Plan” [81] compiled in Poland identifies areas at the greatest risk of drought, including hydrological drought, however it does not address the most adverse maximum droughts in terms of economic and social effects. In Poland, there is also a monitoring system developed by the Institute of Meteorology and Water Management–National Research Institute (https://meteo.imgw.pl/?model=hybrid&loc=warszawa_/warszawa&ter=1465&mode=details accessed on 30 July 2022) which provides information on warnings where rivers have a streamflow below the average low flow from a multi-annual period, as well as the Agricultural Drought Monitoring System developed by a research team from the Puławy Institute (maps of South-eastern Climate Water Balance and maps of the potential extent of agricultural drought), (https://susza.iung.pulawy.pl/ accessed on 30 July 2022).

Unfortunately, as of yet there is no specific system of early drought warning, including extreme drought. In 2021 in Poland, the “Water Shortage Counteracting Programme” was developed [82] with its primary aim to increase water retention in Poland. The programme is currently at the legislative stage and in the course of intra-ministerial consultations. The document is set to be adopted by the Council of Ministers by the end of 2022.

Droughts, especially extreme ones, are one of the natural disasters which affect human life, and the long-term shortage of water causes damage to societies and the economy. This is why a comprehensive method of drought monitoring is indispensable to identify the cases of drought as part of the policies of early warning and mitigation of effects.

The results of this work, concerning drought characteristics at a regional level, may increase the competencies of decision-makers, enabling them to develop better planning and strategies for mitigating the effects of drought. The proposed method of monitoring droughts with a specific duration and volume exceeding the set values in a given region in a year and in a given area is applied by utilising a bivariate analysis based on a copula for different drought characteristics. An analysis of the maps which present spatial distributions of maximum drought frequency of occurrence also allows for the determination of areas in the Polish Carpathians more or less at risk of annual maximum drought of duration and/or volume exceeding a given value. A large number of studies have examined the characteristics of droughts in the Polish Carpathians; however, as of yet no-one has studied the hydrological drought hazard in the Polish Carpathians from the perspective of bivariate probability distributions. The information obtained may be used in the future planning of water management and the mitigating of drought effects in the Polish Carpathians.

## Figures and Tables

**Table 1:** Characteristic of gauging cross-sections and catchments enclosed by those sections and percentile Q_70%_ read read from the flow duration curve, in the 30-year period (1991–2020).

	River\Gauging Station	Latitude (N)	Longtitude (E)	Catchment Area (km^2^)	Gauging Station Elevation (m a.s.l.)	*Q*_70%_ (m^3^/s)
1	Wisła\Wisła	49°37′55″	18°54′03″	53.6	470.6	0.42
2	Wisła\Ustroń-Obłaziec	49°40′50″	18°51′01″	107.4	398.7	0.94
3	Biała\Mikuszowice	49°46′46″	19°04′27″	32.6	360.9	0.28
4	Soła\Rajcza	49°30′50″	19°06′59″	253.8	482.0	1.67
5	Soła\Żywiec	49°41′11″	19°11′36″	782.8	342.0	5.36
6	Żabniczanka\Żabnica	49°33′53″	19°10′49″	23.9	564.8	0.22
7	Skawa\Jordanów	49°38′11″	19°49′48″	96.8	442.9	0.29
8	Skawa\Osielec	49°41′01″	19°44′21″	243.6	393.6	1.04
9	Skawica\Zawoja	49°38′26″	19°32′07″	46.1	577.5	0.56
10	Skawica\Skawica Dolna	49°41′10″	19°39′49″	135.7	408.0	1.27
11	Stryszawka\Sucha Beskidzka	49°44′36″	19°36′04″	140.5	323.9	0.65
12	Skawinka\Radziszów	49°56′24″	19°48′32″	318.1	213.5	0.94
13	Raba\Kasinka Mała	49°42′17″	20°01′57″	353.3	356.9	2.04
14	Raba\Stróża	49°47′49″	19°55′29″	644.2	297.0	3.62
15	Czarny Dunajec\Koniówka	49°23′44″	19°49′05″	132.8	725.8	2.00
16	Dunajec\Czchów	49°48′59″	20°40′55″	5317.3	222.4	32.30
17	Białka\Łysa Polana	49°15′49″	20°06′54″	63.4	965.6	1.08
18	Niedziczanka\Niedzica	49°24′41″	20°18′08″	136.7	495.7	0.74
19	Poprad\Muszyna	49°20′22″	20°53′31″	1518.8	446.3	8.70
20	Poprad\Muszyna-Milik	49°21′00″	20°53′07″	1700.4	440.4	9.92
21	Poprad\Stary Sącz	49°34′07″	20°39′35″	2075.0	295.3	12.00
22	Kamienica\Łabowa	49°31′35″	20°51′32″	64.9	450.2	0.32
23	Kamienica\Nowy Sącz	49°37′31″	20°41′45″	237.0	278.8	1.11
24	Łososina\Jakubkowice	49°44′22″	20°37′48″	347.1	246.3	1.35
25	Biała\Grybów	49°37′26″	20°56′45″	207.0	320.5	0.62
26	Biała\Ciężkowice	49°47′32″	20°58′25″	524.6	238.5	1.55
27	Biała\Koszyce Wielkie	49°59′50″	20°56′58″	955.0	189.7	3.10
28	Wisłoka\Żółków	49°43′48″	21°27′33″	582.0	224.9	1.90
29	Wisłoka\Krajowice	49°46′20″	21°24′45″	2095.4	213.4	7.76
30	Wisłoka\Łabuzie	49°59′16″	21°18′35″	2552.7	184.7	9.92
31	Ropa\Topoliny	49°43′29″	21°26′36″	974.2	224.8	4.41
32	Sękówka\Gorlice	49°39′17″	21°10′13″	122.5	279.2	0.46
33	Jasiołka\Zboiska	49°34′28″	21°41′54″	264.3	311.6	0.94
34	San\Zatwarnica	49°14′06″	22°33′47″	494.2	486.2	3.69
35	San\Dynów	49°48′03″	22°14′39″	2944.9	234.8	19.40
36	Czarna\Polana	49°18′09″	22°34′27″	94.1	437.4	0.58
37	Solinka\Terka	49°17′59″	22°25′45″	309.1	432.8	2.67
38	Wetlina\Kalnica	49°11′20″	22°25′45″	119.0	573.7	1.17
39	Wisłok\Żarnowa	49°52′40″	21°49′03″	1433.0	213.5	4.68
40	Morwawa\Iskrzynia	49°40′38″	21°51′51″	107.4	266.3	0.35

**Table 2:** Selected copulas $C_{\theta }\left(t,v\right)$ [based on 69].

Copula	$C_{\theta }^{}\left(t,v\right)$
Archimedean, Clayton	$\max\;\left({\left[t^{-\theta }+v^{-\theta }-1\right]}^{-1/\theta },0\right),\;\theta \in [-1,\;+\infty )\backslash \{0\}$
Archimedean, Frank	$-\frac{1}{\theta }\ln\left(1+\frac{\left(e^{-\theta \;t}-1\right)\left(e^{-\theta \;v}-1\right)}{e^{-\theta }-1}\right),\;\theta \in (-\infty ,\;+\infty )\backslash \{0\}$
Archimedean, Gumbel–Hougaard	$\exp\left\{-{\left[{\left(-\ln t\right)}^{\theta }+{\left(-\ln v\right)}^{\theta }\right]}^{\frac{1}{\theta }}\right\},\;\theta \in [1,\;+\infty )$
Plackett	$\{\begin{array}{ll} \frac{1+(\theta -1)(t+v)-\sqrt{{\left[1+(\theta -1)(t+v)\right]}^{2}-4\theta (\theta -1)\;tv}}{2(\theta -1)}, & \theta \in (0,\;+\infty )\backslash \{1\}\\ uv, & \theta =1 \end{array}$

**Table 3:** *p_v_*-values of of the bivariate Anderson–Darling goodness-of-fit test (* means *p_v_*-values greater than 0.05), Kendall τ and Spearman ρ correlation coefficients of the variables $T_{max}^{g}$ and $V_{max}^{g}$ computed for the probability distribution by using Gumbel–Hougaard copula, Gumbela-Hougaarda copula parameter and return period *T_p_* droughts (*T_max_*_,10_, *V_max_*_,10_) and $\left({\bar{T}}_{max},{\bar{V}}_{max}\right)$.

No.	River\Gauging Station	***p_v_*** **Cl**	***p_v_*** **Fr**	***p_v_*** **GH**	***p_v_*** **Pl**	**The Best Copula**	**τ** **GH**	**ρ** **GH**	***θ*** **GH**	***T_p_*** $\left({\bar{T}}_{max},{\bar{V}}_{max}\right)\;$	***T_p_*** **(*T_max_*_,10_,*V_max_*_,10_)**
1	Wisła\Wisła	0.074 *	0.184 *	0.294 *	0.150 *	GH	0.951	0.935	4.548	2.90	12.24
2	Wisła\Ustroń-Obłaziec	0.001	0.054 *	0.148 *	0.040	GH	0.958	0.937	4.780	2.97	12.88
3	Biała\Mikuszowice	0.055 *	0.038	0.104 *	0.062 *	GH	0.506	0.417	1.400	3.10	15.64
4	Soła\Rajcza	0.041	0.149 *	0.152 *	0.069 *	GH	0.921	0.900	3.661	2.74	11.67
5	Soła\Żywiec	0.021	0.131 *	0.270 *	0.131 *	GH	0.958	0.950	4.924	2.70	11.66
6	Żabniczanka\Żabnica	0.001	0.143 *	0.238 *	0.067 *	GH	0.904	0.873	3.239	2.87	11.72
7	Skawa\Jordanów	0.009	0.120 *	0.205 *	0.062 *	GH	0.923	0.896	3.626	2.88	12.46
8	Skawa\Osielec	0.001	0.049	0.120 *	0.050 *	GH	0.926	0.896	3.642	2.90	12.45
9	Skawica\Zawoja	0.092 *	0.296 *	0.297 *	0.231 *	GH	0.868	0.826	2.809	2.82	12.08
10	Skawica\Skawica Dolna	0.002	0.083 *	0.228 *	0.055 *	GH	0.907	0.875	3.266	2.88	11.73
11	Stryszawka\Sucha Beskidzka	0.022	0.124 *	0.166 *	0.140 *	GH	0.868	0.815	2.724	2.92	12.43
12	Skawinka\Radziszów	0.041	0.128 *	0.129 *	0.099 *	GH	0.955	0.938	4.738	2.81	13.54
13	Raba\Kasinka Mała	0.004	0.048	0.161 *	0.026	GH	0.957	0.938	4.763	3.17	12.84
14	Raba\Stróża	0.032	0.054 *	0.133 *	0.041	GH	0.969	0.953	5.552	2.96	13.71
15	Czarny Dunajec\Koniówka	0.064 *	0.153 *	0.146 *	0.177 *	Pl	0.957	0.941	4.886	2.91	12.58
16	Biały Dunajec\Szaflary	0.028	0.089 *	0.110 *	0.095 *	GH	0.957	0.935	4.615	2.93	11.80
17	Białka\Łysa Polana	0.012	0.100 *	0.197 *	0.075 *	GH	0.937	0.913	4.073	3.00	12.11
18	Niedziczanka\Niedzica	0.018	0.059 *	0.09 *	0.057 *	GH	0.932	0.911	3.952	2.94	12.19
19	Poprad\Muszyna	0.012	0.098 *	0.178 *	0.079 *	GH	0.963	0.952	5.490	3.34	11.78
20	Poprad\Muszyna-Milik	0.005	0.094 *	0.201 *	0.087 *	GH	0.953	0.935	4.754	2.90	11.73
21	Poprad\Stary Sącz	0.008	0.067 *	0.108 *	0.049	GH	0.966	0.958	5.772	2.79	11.37
22	Kamienica\Łabowa	0.000	0.112 *	0.236 *	0.132 *	GH	0.936	0.912	4.059	2.91	12.00
23	Kamienica\Nowy Sącz	0.022	0.081 *	0.185 *	0.069 *	GH	0.943	0.921	4.238	2.82	12.12
24	Łososina\Jakubkowice	0.005	0.105 *	0.113 *	0.048	GH	0.939	0.917	4.120	2.79	11.43
25	Biała\Grybów	0.003	0.098 *	0.199 *	0.077 *	GH	0.901	0.874	3.288	2.84	12.82
26	Biała\Ciężkowice	0.002	0.077 *	0.140 *	0.081 *	GH	0.946	0.924	4.300	3.00	12.38
27	Biała\Koszyce Wielkie	0.000	0.057 *	0.116 *	0.042	GH	0.938	0.918	4.031	3.07	12.14
28	Wisłoka\Żółków	0.007	0.077 *	0.143 *	0.072 *	GH	0.946	0.924	4.366	2.77	11.51
29	Wisłoka\Krajowice	0.084 *	0.182 *	0.191 *	0.128 *	GH	0.960	0.950	5.322	2.81	11.93
30	Wisłoka\Łabuzie	0.036	0.101 *	0.176 *	0.067 *	GH	0.946	0.936	4.607	2.85	11.80
31	Ropa\Topoliny	0.155 *	0.261 *	0.228 *	0.283 *	Pl	0.900	0.866	3.211	3.43	13.36
32	Sękówka\Gorlice	0.101 *	0.155 *	0.159 *	0.159 *	GH	0.909	0.870	3.333	3.04	12.76
33	Jasiołka\Zboiska	0.101 *	0.155 *	0.159 *	0.159 *	GH	0.909	0.870	3.333	2.74	12.76
34	San\Zatwarnica	0.003	0.029	0.088 *	0.009	GH	0.969	0.956	5.788	2.77	11.36
35	San\Dynów	0.129 *	0.163 *	0.114 *	0.189 *	Pl	0.953	0.936	4.650	2.71	11.45
36	Czarna\Polana	0.000	0.013	0.066 *	0.009	GH	0.930	0.902	3.809	2.76	11.49
37	Solinka\Terka	0.004	0.086 *	0.135 *	0.059 *	GH	0.963	0.948	5.278	2.57	11.52
38	Wetlina\Kalnica	0.037	0.111 *	0.248 *	0.119 *	GH	0.971	0.959	5.919	2.69	11.33
39	Wisłok\Żarnowa	0.023	0.096 *	0.185 *	0.084 *	GH	0.965	0.952	5.374	2.92	12.30
40	Morwawa\Iskrzynia	0.000	0.208 *	0.157 *	0.118 *	Fr	0.951	0.933	4.682	2.94	12.17

**Table 4:** Color and linguistic coding in the adopted *T_P_* quartile classification of the frequency of occurrence of the maximum annual droughts (*T_max_* > *x*, *V_max_* > *y*), (*x*,*y*) = (*T_max_*_,10_, *V_max_*_,10_) i $\left({\bar{T}}_{max},{\bar{V}}_{max}\right)$.

Cathegory	The Color Assigned to the Category	The Frequency of the Maximum Drought (*T_max_* > *x*, *V_max_* > *y*)	The Return Period *T_P_* Oft he Maximum Drought (*T_max_* > *x*, *V_max_* > *y*)	Maximum Drought Hazard (*T_max_* > *x*, *V_max_* > *y*)
((1/*T_P_*)_75%_; (1/*T_P_*)*_max_*]		the lowest	the longest	the lowest
((1/*T_P_*)_50%_; (1/*T_P_*)_75%_]		moderate	long	moderate
((1/*T_P_*)_25%_; (1/*T_P_*)_50%_]		high	moderate	high
[(1/*T_P_*)*_min_*; (1/*T_P_*)_25%_]		the highest	the shortest	the highest
